# Identification of Differentially Expressed Intronic Transcripts in Osteosarcoma

**DOI:** 10.3390/ncrna8060073

**Published:** 2022-10-25

**Authors:** Emel Rothzerg, Jiake Xu, David Wood

**Affiliations:** 1 School of Biomedical Sciences, The University of Western Australia, Perth, WA 6009, Australia; 2 Medical School, The University of Western Australia, Perth, WA 6009, Australia

**Keywords:** osteosarcoma, intronic transcript, lncRNA, micropeptide, differential expression

## Abstract

Over the past decade; the discovery and characterization of long noncoding RNAs (lncRNAs) have revealed that they play a major role in the development of various diseases; including cancer. Intronic transcripts are one of the most fascinating lncRNAs that are located within intron regions of protein-coding genes, which have the advantage of encoding micropeptides. There have been several studies looking at intronic transcript expression profiles in cancer; but almost none in osteosarcoma. To overcome this problem; we have investigated differentially expressed intronic transcripts between osteosarcoma and normal bone tissues. The results highlighted that NRG1-IT1; FGF14-IT1; and HAO2-IT1 were downregulated; whereas ER3-IT1; SND1-IT1; ANKRD44-IT1; AGAP1-IT1; DIP2A-IT1; LMO7DN-IT1; SLIT2-IT1; RNF216-IT1; and TCF7L1-IT1 were upregulated in osteosarcoma tissues compared to normal bone tissues. Furthermore, we identified if the transcripts encode micropeptides and the transcripts’ locations in a cell.

## 1. Introduction

Osteosarcoma (OS) is the most common primary solid tumour of bone in children and adolescents with a median age of 16 years [1]. The incidence of the tumour is common in the metaphyseal area of long bones including the distal femur, the proximal humerus, and the proximal tibia. OS also may develop in other parts of the skeleton including the spine, and pelvis [2]. It is a highly aggressive tumour type with a propensity for local invasion and systematic early metastasis to the lungs [3]. The current treatment of OS is a combination of limb-salvage surgery, neoadjuvant and adjuvant chemotherapy [4]. The exact aetiology of OS is still unknown, but there are several risk factors (such as genetic factors) that may have an association with the development and progression of the disease [5].

The Human Genome Project has revealed that less than 2% of the human genome was protein-coding [6]. Protein-coding genes have become the major research focus for decades, while non-coding RNAs (ncRNA) were considered as the transcriptional noise and sometimes referred to as junk RNA because they did not contain open reading frames (ORFs) [7,8]. However, advanced computer tools and high-throughput sequencing techniques have reported that many ncRNAs contain short ORFs (sORFs). The products encoded by sORFs are named micropeptides, with a length of less than 100 amino acids [9,10,11]. Emerging evidence indicates that micropeptides regulate several biological processes including cell proliferation, cell death, and myogenesis [11]. Generally, ncRNAs are classified into two groups based on their nucleotide (nt) length; short ncRNAs (sncRNA) are less than 200 nt in length and long ncRNAs (lncRNA) are more than 200 nt [8]. Intronic transcripts (ITs) are one of the critical lncRNAs that are located within intron regions of protein-coding genes which have the potential to encode micropeptides.

The role of ITs in the genesis and progression of osteosarcoma has been poorly investigated. To overcome this issue, we have investigated the differential expression of ITs in OS samples compared to normal bone samples using RNA sequencing (RNA-seq).

## 2. Results

A total of 45 OS patients between the age of 10 and 78 participated in this study; 24 patients received chemotherapy with wide resection, 12 patients received chemotherapy alone, 5 patients received chemotherapy with an above-the-knee amputation (AKA), 2 patients received wide resection alone, and 2 patients received no treatment including no chemotherapy. Out of 45 patients, 22 of them were alive with no evidence of the disease, 19 of them died from the disease, 3 them alive with metastasis of the disease, and 1 was unknown (Table 1). 

**Figure 1:**
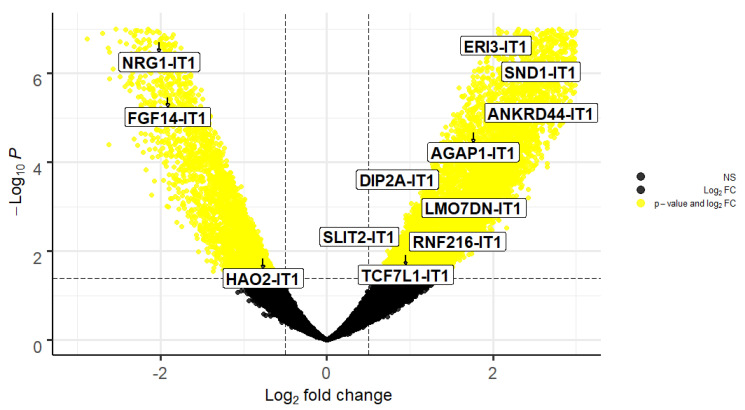
Volcano plot of the distributions of differentially expressed genes and transcripts. The yellow dots indicate the differentially expressed genes and transcripts (adjusted *p* < 0.05) between osteosarcoma tumour versus normal bone samples. The block dots represent not significant differentially expressed genes ad transcripts. The vertical and horizontal dotted lines highlight the cut-off value of Log2 fold-change = ±0.5, and of *p*-value (−Log_10_p) = 0.05, respectively. The plot indicates that NRG1-IT1, FGF-IT1 and HAO2-IT1 were downregulated in osteosarcoma tumour samples compared to normal, whereas ERI3-IT1, SND1-IT1, ANKRD44-IT1, AGAP1-IT1, DIP2A-IT1, LMO7DN-IT1, SLIT2-IT1, RNF216-IT1, and TCF7L1-IT1 were upregulated in tumour samples. The volcano plot was generated using R studio software (version 4.1.0).

The DETs between OS and normal bone samples were obtained through the DESeq2 package. We have identified 12 statistically significant DETs between the OS and normal bone samples; Neuregulin 1-IT1 (NRG1-IT1), Fibroblast Growth Factor 14-IT1 (FGF14-IT1), Hydroxyacid Oxidase 2-IT1 (HAO2-IT1) were downregulated in tumour samples, whereas Exoribonuclease Family Member 3-IT1 (ERI3-IT1), Staphylococcal Nuclease Additionally, Tudor Domain Containing 1-IT1 (SND1-IT1), Ankyrin Repeat Domain 44-IT1 (ANKRD44-IT1), ArfGAP With GTPase Domain, Ankyrin Repeat Additionally, PH Domain 1-IT1 (AGAP1-IT1), Disco Interacting Protein 2 Homolog A-IT1 (DIP2A-IT1), LMO7 Downstream Neighbor-IT1 (LMO7DN-IT1), Slit Guidance Ligand 2-IT1 (SLIT2-IT1), Ring Finger Protein 216-IT1 (RNF216-IT1), Transcription Factor 7 Like 1-IT1 (TCF7L1-IT1), and Hydroxyacid Oxidase 2-IT1 (HAO2-IT1) were upregulated in tumour samples compared to normal bone samples (Figure 1 and Table 2).

**Figure 2:**
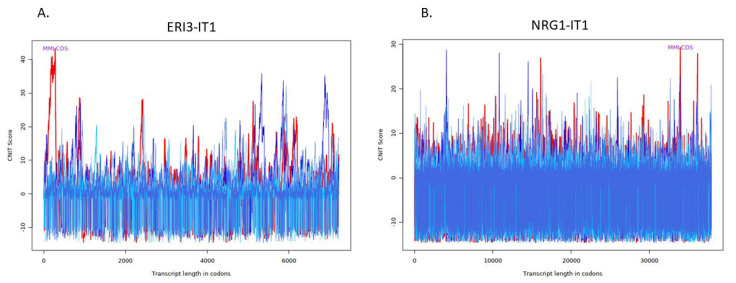

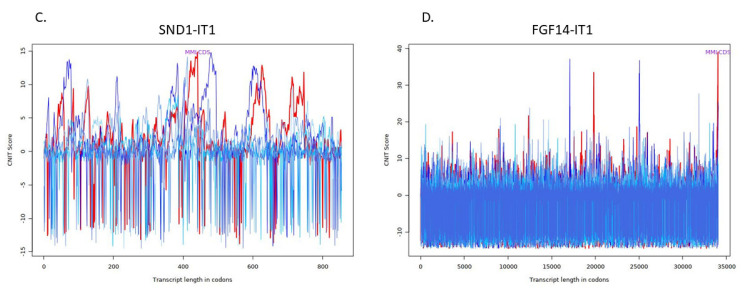

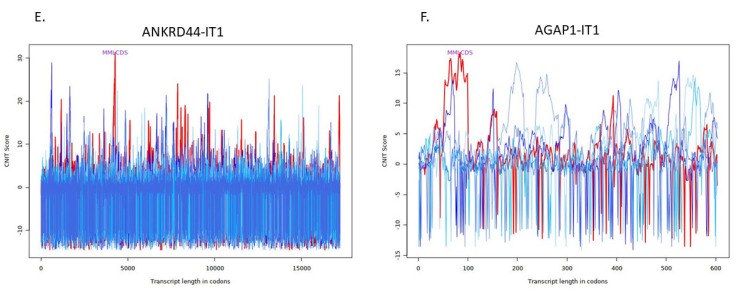

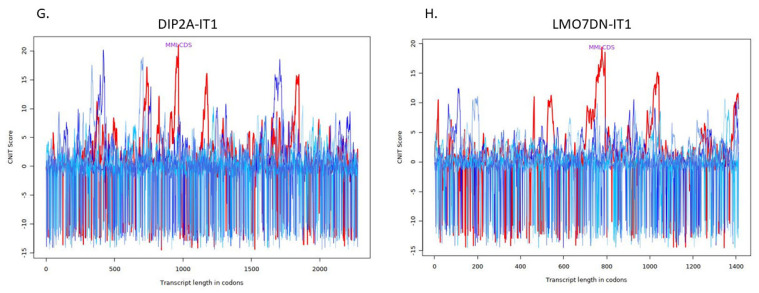

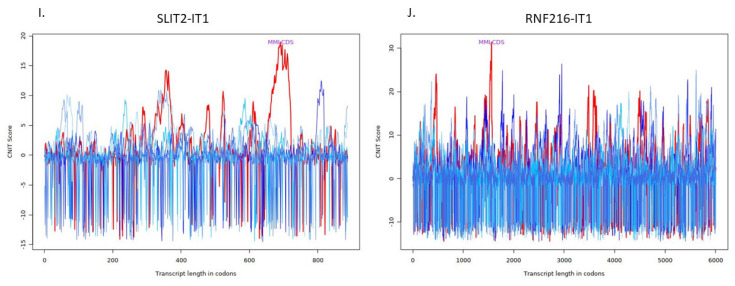

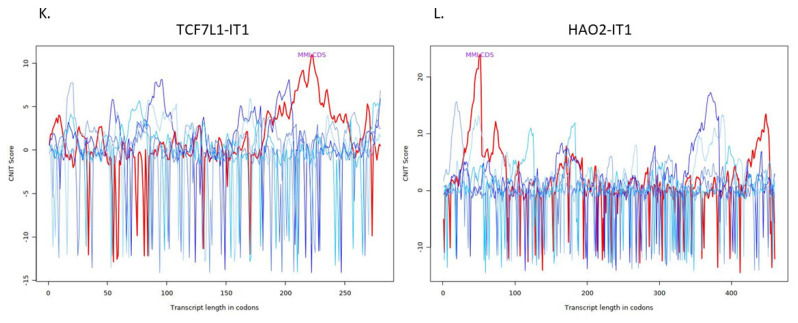
CNIT Score Detail Plots of (**A**) ERI3-IT1, (**B**) NRG1-IT1, (**C**) SND1-IT1, (**D**) FGF14-IT1, (**E**) ANKRD44-IT1, (**F**) AGAP1-IT1, (**G**) DIP2A-IT1, (**H**) LMO7DN-IT1, (**I**) SLIT2-IT1, (**J**) RNF216-IT1, (**K**) TCF7L1-IT1, and (**L**) HAO2-IT1. Red line represents the correct transcriptional reading frame and other five lines (blue or green) represent other five reading frames. Green line highlights the distribution of the coverage (the right y-axis) of the most-like coding domain sequence (MLCDS) region for each transcript across the normalized length. The x axis indicates transcript length in codons, whereas y axis indicates CNIT score. The total length of the identified sequence of transcripts converted to codons length (the identified sequence length/3).

To understand the transcripts’ role in tumour development we further investigated which transcripts have the potential to encode proteins. The CNIT scores highlighted that NRG1-IT1, FGF14-IT1, and ANKRD44-IT1 encode micropeptides (Figure 2 and Table 3). Further, the LncLocator tool showed that ERI3-IT1 and SND1-IT1 locate in the nucleus, NRG1-IT1, FGF14-IT1, ANKRD44-IT1, LMO7DN-IT1, SLIT2-IT1, TCF7L1-IT1, HAO2-IT1 locate in the cytoplasm, AGAP1-IT1 and DIP2A-IT1 locate in cytosol and RNF216-IT1 locates in the ribosome (Table 3). 

**Figure 3:**
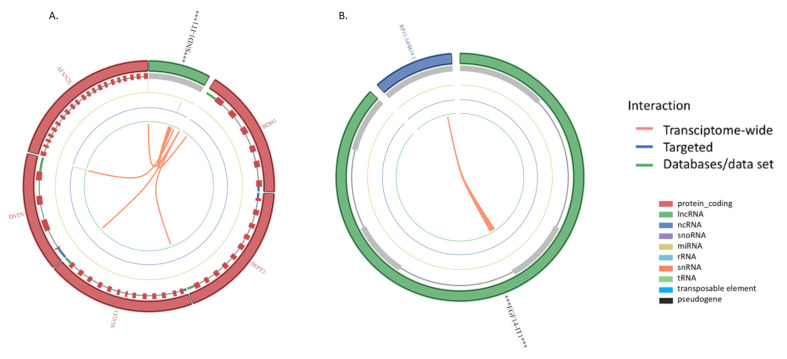

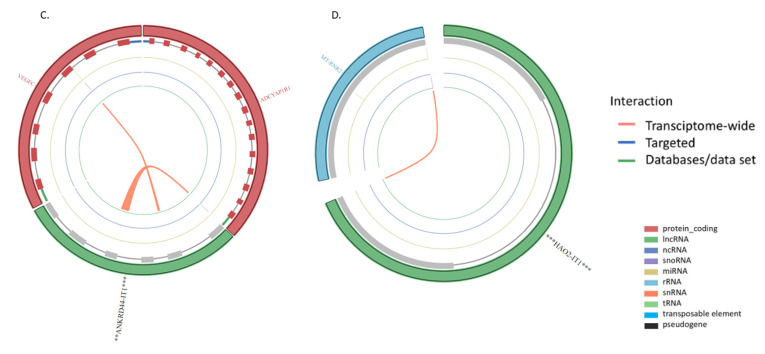
The circos plots show the lncRNA-RNA interaction of (**A**) SND1-IT1, (**B**) FGF14-IT1, (**C**) ANKRD44-IT1, and (**D**) HAO2-IT1. The ribbon colours highlight the interaction of the intronic transcripts with other transcripts and genes such as Transcriptome-wide (orange), Targeted (blue) and Databases/data set (green). Transcript colours; protein-coding (red), lncRNA (green), and ncRNA (blue).

Finally, we have investigated lncRNA- lncRNA and lncRNA-RNA interactions using the RISE tool. According to the results, various interactions between lncRNA- lncRNA and lncRNA-RNA are still unknown and need to be investigated. However, the circos plot in Figure 3A highlights that SND1-IT1 interacts with Ataxin 2 like (*ATXN2L*), Destrin (*DSTN*), SGT1 Homolog, MIS12 Kinetochore Complex Assembly Cochaperone (*SUGT1*), Septin 3 (*SEPT3*), and D-beta-hydroxybutyrate dehydrogenase (BDH1). FGF14-IT1 interacts with RP11-549B18.1 (Figure 3B). ANKRD44-IT1 interacts with *ADCYAP* receptor type I (*ADCYAP1R1*) and vascular endothelial growth factor-C (*VEGFC*) (Figure 3C). HAO2-IT1 interacts with *MT-RNR2* (Figure 3D).

## 3. Discussion

The field of ncRNAs is growing in cancer genomics and precision oncology. Recently, it has been found that several lncRNAs are involved in tumorigenesis. Although the functions of lncRNAs in OS occurrence and progression remain an emerging field, only a handful lncRNAs are known to be functional in OS development such as MALAT1, TUG1, XIST, and PVT1 [12,13,14]. Disappointingly, out of approx. 55,000 ITs, only SPRY4-IT1 and SND1-IT1 association with OS have been studied [15,16,17,18].

In the present study, we specifically investigated DETs between OS and normal samples. The results highlighted that NRG1-IT1, FGF14-IT1, and HAO2-IT1 were downregulated in OS samples, whereas ER3-IT1, SND1-IT1, ANKRD44-IT1, AGAP1-IT1, DIP2A-IT1, LMO7DN-IT1, SLIT2-IT1, RNF216-IT1, and TCF7L1-IT1 were upregulated in OS samples compared to normal tissue controls. 

SND1-IT1 is one of the newly discovered ITs that regulates OS development and progression. It was found that knockdown of SND1-IT1 reduced cell proliferation and migration in OS cells [18]. The transcript also was upregulated in the OS samples compared to normal (Figure 1 and Table 2). The results also showed that SND1-IT1 is expressed in the nucleus of a cell without the function of encoding micropeptides (Table 3). The lncRNAs that localise in the nucleus can modulate epigenetic regulation, phase separation, and chromatin function and alter the stability and translation of mRNA, further disrupting signal-transduction pathways [19,20]. Interestingly, SND1-IT1 accelerates cell proliferation, migration, and invasion in retinoblastoma [21]. The transcript also plays a role in epithelial-mesenchymal transition in gastric cancer [22]. Our results showed that SND1-IT1 interacts with ATXN2L, DSTN, SUGT1, SEPT3, and BDH1 genes. To date there are no reported associations between OS development and ATXN2L, DSTN, SUGT1, SEPT3, and BDH1 genes. However, BDH1 expression is linked with liver cancer [23], acute myeloid leukaemia [24], and hepatocellular carcinoma [25]. In addition, ATXN2L expression was upregulated by epidermal growth factor which promotes gastric cancer cell invasion and drug resistance [26]. High expression of SUGT1 is linked with human colorectal cancer [27].

SLIT2-IT1 is regulated by the SLIT2 promoter hyper-methylation during myelodysplastic neoplasm progression in leukemia, further high expression of the transcript increases cell proliferation, cell mitosis rate, colony formation, and apoptosis resistance in leukemogenesis [28].

Unfortunately, there is a dearth of literature on NRG1-IT1, HAO2-IT1, ER3-IT1, SND1-IT1, ANKRD44-IT1, AGAP1-IT1, DIP2A-IT1, LMO7DN-IT1, SLIT2-IT1, RNF216-IT1, and TCF7L1-IT1 in tumorigenesis.

In the present study, we also observed that NRG1-IT1, FGF14-IT1, and ANKRD44-IT1 encode micropeptides (Table 3). NRG1-IT1 and FGF14-IT1 were both downregulated in OS samples, whereas ANKRD44-IT1 was upregulated. According to our results, FGF14-IT1 interacts with the RP11-549B18.1 transcript. Interestingly, the RP11-549B18.1 transcript and its variants were associated with Alzheimer’s disease in a Genome-Wide Association Study [29]. We also observed that ANKRD44-IT1 interacts with VEGFC and ADCYAP1R1. Crucial functions of VEGFC enhance cancer cell mobility and increase invasion capabilities in solid tumours, consequently, promoting cancer cell metastasis to distant sites through lymphangiogenesis [30,31]. Expression of VEGFC and its receptor were found in OS samples [32], further, it was suggested that overexpression of VEGFC regulates angiogenesis in OS [33,34]. The findings imply that overexpression of ANKRD44-IT1 which encodes miropeptides in the cytoplasm of a cell may be associated with OS progression.

In conclusion, this study confirms that lncRNA ITs play a role in OS development and progression. We have investigated differential IT expression in OS samples compared to normal bone tissues. The results suggested that 3 ITs (NRG1-IT1, FGF14-IT1, and HAO2-IT1) were downregulated in OS samples and 9 ITs (ER3-IT1, SND1-IT1, ANKRD44-IT1, AGAP1-IT1, DIP2A-IT1, LMO7DN-IT1, SLIT2-IT1, RNF216-IT1, and TCF7L1-IT1) were upregulated in OS samples compared with normal bone tissues. Unfortunately, the transcripts were poorly characterized and have not been studied in cancer, especially in OS. Further work is required to understand the role of ITs in OS development and progression.

## 4. Materials and Methods

### 4.1. Sample Description 

This study has been approved by the Human Research Ethics Review Committee of the University of Western Australia and Sir Charles Gairdner Hospital (2019/RA/4/20/5211). The patient informed consent forms were signed and personally dated by the participants and by the participant’s legally acceptable representatives before the limb-sparing or amputation surgery.

Forty-five Australian OS patients underwent the surgery to remove the tumour and surrounding normal tissue. Cancerous (*n* = 45) and normal bone tissue (tissue surrounding the tumour) (*n* = 40) formalin-fixed paraffin-embedded (FFPE) tissue samples were collected from PathWest (QEII Medical Centre, Nedlands, WA, Australia).

### 4.2. Total RNA Isolation and Sequencing 

Total RNA was extracted from recently cut 5 sections of ≤20 µm thick FFPE sections using the FFPE RNA purification kit (Norgen Biotek, Thorold, ON, Canada), according to the manufacturer’s instructions. RNA yield and quality of total RNA were measured by the NanoDrop™ One/OneC Microvolume UV-Vis Spectrophotometer (ThermoFisher Scientific, Waltham, MA, USA) and fragment size was analysed using the RNA 6000 Nano Kit (Agilent Technologies Inc., Santa Clara, CA, USA) run on the 2100 Bioanalyzer. 

The RNA samples were prepared for sequencing using the Takara SMARTer V2 Total RNA Mammalian Pico Input protocol using 2 ng of Total RNA input as per the manufacturer’s protocol (Takara Bio Inc., Mountain View, CA, USA). The libraries were sequenced on Illumina NovaSeq 6000 and an S4-300 cycle lane (150PE) with v1.5 sequencing chemistry [35]. The quality score distribution of the sequencing was obtained by the FastQC quality control tool (version 0.11.9). The low-quality reads (PHRED score < 20 and read length < 25 bp) were trimmed and filtered out using Trimmomatic (version 0.39) [36,37]. RNA-seq reads were aligned to the human reference genome hg38 (GRCH38) using STAR (version 2.7.7a) [38].

### 4.3. Identification of Differentially Expressed Transcripts

In this study we used DESeq2 to screen differentially expressed transcripts (DETs) between OS tissue and normal bone samples. The Raw counts were normalised using the transcripts per million (TPM) method. The Benjamini–Hochberg approach was used to adjust the *p*-value (padj) by the Deseq2 package. The screening conditions were the logarithmic-2-fold changes with the cut-off value of 0.5 and padj < 0.05. The DETs were visualised using the EnhancedVolcano package through R studio. 

### 4.4. Investigation of Structure and Function of lncRNAs 

The Coding-Non-coding Identifying Tool (CNIT) was used to identify the coding potential of the transcripts [39]. The subcellular localization of lncRNAs was investigated using the LncLocator tool [40]. Further, the RISE database was used to highlight lncRNAs interactions and networking with other transcripts [41].

## Figures and Tables

**Table 1:** Characteristics of osteosarcoma patients in the present study.

Patient ID	Tumour	Normal	YoB *	AtDi *	Vital status	Treatment Options	AtDe *	Outcome
Q18B006524D	A28	A8	1944	74	Dead	No chemotherapy	74	Died from the disease
Q13B004130D	A9	A3	1990	22	Dead	Chemotherapy and wide resection	26	Died from the disease
Q17B001640B	B5	B1	1992	24	Alive	Chemotherapy and wide resection		Alive with no evidence of the disease
Q10B040965M	A4	A55	1990	20	Alive	Chemotherapy and wide resection		Alive with no evidence of the disease
Q16B040208X	A33	A25	1999	17	Dead	Chemotherapy and wide resection	18	Died from the disease
Q19B013567K	A1	-	1956	63	Dead	Chemotherapy and wide resection	64	Died from the disease
Q18B028621H	A4	A1	1992	26	Alive	Chemotherapy and wide resection		Alive with no evidence of the disease
Q09B042936F	B21	B9	1992	17	Alive	Chemotherapy and wide resection		Alive with no evidence of the disease
Q08B047467N	A18	A3	1994	14	Alive	Chemotherapy and wide resection		Alive with no evidence of the disease
Q11B045903J	A5	A31	1985	25	Alive	Chemotherapy and wide resection		Lost to follow up
Q14B020064N	A9	A1	1990	24	Alive	Chemotherapy and AKA*		Alive with no evidence of the disease
Q05B030211M	A29	A30	1988	17	Alive	Chemotherapy and AKA		Alive with no evidence of the disease
Q18B014955A	A15	A23	2001	17	Dead	Chemotherapy and wide resection	18	Died from the disease
Q19B005830Y	A2	-	2002	17	Alive	Chemotherapy and wide resection		Alive with metastasis of the disease
Q16B027819Y	A23	A16	1995	17	Alive	Chemotherapy and wide resection		Alive with no evidence of the disease
Q19B001229R	A30	A22	2005	13	Alive	Chemotherapy and wide resection		Alive with no evidence of the disease
Q01B033022A	B2	A1	1985	16	Dead	Chemotherapy and AKA	17	Died from the disease
Q12B042591T	A1	-	1995	16	Dead	Chemotherapy and wide resection	18	Died from the disease
Q15B001034Y	A15	B1	1995	19	Dead	Chemotherapy	20	Died from the disease
Q04B025963T	B28	A1	1988	16	Alive	Chemotherapy AKA		Alive with no evidence of the disease
Q17B009637R	A2	B1	1997	19	Alive	Chemotherapy and wide resection		Alive with no evidence of the disease
Q17B018941H	A12	B1	1981	36	Dead	Chemotherapy	37	Died from the disease
Q17B029593M	A7	A23	1994	23	Dead	Chemotherapy	26	Died from the disease
Q18B051017F	A1	A16	1949	69	Dead	Chemotherapy	71	Died from the disease
Q02B032169Y	B18	B12	1988	14	Dead	Chemotherapy and AKA	17	Died from the disease
Q05B009812W	A29	A32	1983	21	Alive	Chemotherapy and wide resection		Alive with no evidence of the disease
Q19B051495P	B19	A2	2005	14	Alive	Chemotherapy		Alive with no evidence of the disease
Q19B052024A	B2	B6	1983	36	Alive	Wide resection and no chemotherapy		Alive with no evidence of the disease
Q17B045995J	A5	A29	1939	78	Dead	No chemotherapy	80	Died from the disease
Q12B019249N	A28	A34	1992	20	Dead	Chemotherapy and wide resection	21	Died from the disease
Q17B034037Y	B20	B2	1996	21	Dead	Chemotherapy	22	Died from the disease
Q19B035672T	A1	-	1986	33	Alive	Chemotherapy		Alive with no evidence of the disease
Q18B034715Y	A6	A11	1999	19	Alive	Chemotherapy		Alive with metastasis of the disease
Q13B008611L	A13	A5	1997	15	Alive	Chemotherapy and wide resection		Alive with no evidence of the disease
Q12B044305A	A45	A16	1988	26	Alive	Chemotherapy		Alive with no evidence of the disease
Q18B018266Y	A1	E1	1960	58	Dead	Chemotherapy	58	Died from the disease
Q16B037369B	A8	A40	2003	13	Alive	Chemotherapy and wide resection		Alive with no evidence of the disease
Q14B024855K	A15	A29	1997	17	Alive	Chemotherapy and wide resection		Alive with no evidence of the disease
Q19B007088F	B10	B22	2001	17	Alive	Chemotherapy		Alive with metastasis of the disease
Q13B020599E	B5	C3	1997	15	Dead	Chemotherapy and wide resection	18	Died from the disease
Q13B012216B	A21	A7	1993	19	Dead	Chemotherapy and wide resection	24	Died from the disease
Q05B005169W	B7	A1	1995	10	Alive	Chemotherapy and wide resection		Alive with no evidence of the disease
Q13B011918Y	B10	B36	1993	19	Alive	Chemotherapy and wide resection		Alive with no evidence of the disease
Q17B045840Y	A23	A17	2000	17	Alive	Wide resection and no chemotherapy		Alive with no evidence of the disease
Q18B009680H	A1	-	2001	17	Dead	Chemotherapy	18	Died from the disease

* YoB = Year of Birth, * AtDi = Age at Diagnosis, * AtDe = Age at Death, * AKA = Above-the-Knee Amputation, - = absence of the sample.

**Table 2:** The list of differentially expressed intronic transcripts between osteosarcoma tumour and normal bone samples, with their corresponding log2FoldChange, *p*-value, and padj.

ENSEMBL	Gene Name	Symbol	Log2Fold-Change	*p*-Value	padj
ENSG00000233602	ERI3 intronic transcript 1	ERI3-IT1	2.100851605	1.16 × 10^−7^	4.09 x10^−6^
ENSG00000253974	NRG1 intronic transcript 1	NRG1-IT1	−2.018042879	2.05 x 10^−7^	6.41 x10^−5^
ENSG00000279078	SND1 intronic transcript 1	SND1-IT1	2.381895579	1.80 x 10^−6^	3.64 x10^−5^
ENSG00000243319	FGF14 intronic transcript 1	FGF14-IT1	−1.915326152	3.54 x 10^−6^	6.22 x10^−5^
ENSG00000236977	ANKRD44 intronic transcript 1	ANKRD44-IT1	2.03255465	1.52 x10^−5^	0.000197273
ENSG00000235529	AGAP1 intronic transcript 1	AGAP1-IT1	1.762735181	2.18 x10^−5^	0.000262087
ENSG00000223692	DIP2A intronic transcript 1	DIP2A-IT1	1.122484746	0.000484393	0.003011991
ENSG00000223458	LMO7DN intronic transcript 1	LMO7DN-IT1	1.103657774	0.001667561	0.007934539
ENSG00000248228	SLIT2 intronic transcript 1	SLIT2-IT1	0.905390354	0.004889451	0.018302346
ENSG00000237738	RNF216 intronic transcript 1	RNF216-IT1	0.951215789	0.008326804	0.027567856
ENSG00000231134	TCF7L1 intronic transcript 1	TCF7L1-IT1	0.951045146	0.012385694	0.037228194
ENSG00000230921	HAO2 intronic transcript 1	HAO2-IT1	−0.766349343	0.014926443	0.042897412

**Table 3:** The list of differentially expressed intronic transcripts with their condition, CNIT score and their location in a cell.

Transcript	Condition	CNIT Score	Location
ERI3-IT1	Noncoding	−0.2963047740200079	Nucleus
NRG1-IT1	Coding	0.6252927037161629	Cytoplasm
SND1-IT1	Noncoding	−0.40243101938996373	Nucleus
FGF14-IT1	Coding	0.6617306296160084	Cytoplasm
ANKRD44-IT1	Coding	0.6338404271410529	Cytoplasm
AGAP1-IT1	Noncoding	−0.37615561437094636	Cytosol
DIP2A-IT1	Noncoding	−0.3607746484071159	Cytosol
LMO7DN-IT1	Noncoding	−0.36986001298052484	Cytoplasm
SLIT2-IT1	Noncoding	−0.3722801833195233	Cytoplasm
RNF216-IT1	Noncoding	−0.3220773653956067	Ribosome
TCF7L1-IT1	Noncoding	−0.4460247363860751	Cytoplasm
HAO2-IT1	Noncoding	−0.347525626296487	Cytoplasm

## Data Availability

The Coding-Non-coding Identifying Tool (CNIT) = http://cnit.noncode.org/CNIT (accessed on 21 September 2022). The LncLocator tool = http://www.csbio.sjtu.edu.cn/bioinf/lncLocator/ (accessed on 21 September 2022).
